# Fuzzy Functional Dependencies as a Method of Choice for Fusion of AIS and OTHR Data

**DOI:** 10.3390/s19235166

**Published:** 2019-11-26

**Authors:** Medhat Abdel Rahman Mohamed Mostafa, Miljan Vucetic, Nikola Stojkovic, Nikola Lekić, Aleksej Makarov

**Affiliations:** 1 School of Electrical Engineering and Computer Science, European University, 28 Carigradska St., 11000 Belgrade, Serbia; medhat.ma@yahoo.com; 2 Vlatacom Institute, 5 Milutina Milankovica Blv., 11070 Belgrade, Serbia; nikola.lekic@vlatacom.com (N.L.); aleksej@vlatacom.com (A.M.); 3 School of Electrical Engineering, University of Belgrade, Serbia and Vlatacom Institute, 101801 Beograd, Serbia; nikola.stojkovic@vlatacom.com

**Keywords:** HF-OTH radar, AIS, radar tracking, data fusion, fuzzy functional dependencies, maritime surveillance

## Abstract

Maritime situational awareness at over-the-horizon (OTH) distances in exclusive economic zones can be achieved by deploying networks of high-frequency OTH radars (HF-OTHR) in coastal countries along with exploiting automatic identification system (AIS) data. In some regions the reception of AIS messages can be unreliable and with high latency. This leads to difficulties in properly associating AIS data to OTHR tracks. Long history records about the previous whereabouts of vessels based on both OTHR tracks and AIS data can be maintained in order to increase the chances of fusion. If the quantity of data increases significantly, data cleaning can be done in order to minimize system requirements. This process is performed prior to fusing AIS data and observed OTHR tracks. In this paper, we use fuzzy functional dependencies (FFDs) in the context of data fusion from AIS and OTHR sources. The fuzzy logic approach has been shown to be a promising tool for handling data uncertainty from different sensors. The proposed method is experimentally evaluated for fusing AIS data and the target tracks provided by the OTHR installed in the Gulf of Guinea.

## 1. Introduction

An exclusive economic zone (EEZ) is a 200 nmi (approximately 370 km)-wide area which spreads from territorial waters towards the open sea in which a coastal state has exclusive rights to exploit biological and mineral sea resources. The control of this zone posers technological, financial and organizational challenges. As parts of an integrated maritime surveillance (IMS) system, different types of electronic sensors and telecommunication systems can be used for monitoring the zone. However, the range of microwave and optical sensors depends on their working wavelengths and is limited due to atmospheric signal weakening and the Earth’s curvature—distance to horizon. High-frequency (HF) radars operating at decameter wavelengths (3–30 MHz) use vertically polarized surface waves to detect and track targets beyond the horizon, enabling over-the-horizon (OTH) surveillance. Therefore, HF-OTH radars are well suited for EEZ surveillance [1,2].

For effective EEZ tracking, a fusion of data from multiple sensors is needed. In this case, term fusion means integration of data from at least two sensors. Most often, image of maritime situation beyond the horizon is provided by using fusion of data received from HF-OTHR and an automated identification system (AIS) that can be a land-based AIS (LAIS) or a satellite-based AIS (SAIS) [3].

Data fusion received from HF-OTHR and AIS provides extraction of paths which are not confirmed by AIS. This allows for detecting targets that are non-cooperative and which either pose a military threat or conduct illegal activities (such as smuggling) inside the EEZ [4,5].

In this paper we use real data from the HF-OTHR system installed in the Gulf of Guinea obtained with the pertaining detection and tracking software [6,7,8,9], and the current AIS data in order to perform data fusion. It is important to note that the Gulf of Guinea is among the most challenging environments for the targeted application. There are two major reasons for this:

1. HF noise levels in that area are among the highest in world [10], and
2. Absence of a strong regulatory institution (such as European Maritime Safety Agency—EMSA in Europe) sometimes leads to unpredictable behavior of participants in the maritime traffic. This causes a very high latency and questionable quality of AIS data.

Since our goal is fusion of the tracking data provided by different sensors for the same targets, we have employed the fuzzy functional dependency (FFD) concept in order to associate AIS data and OTHR tracks. Methods based on numerical statistics were used in the previous research on AIS and OTHR data fusion [8,11]. However, intensive traffic flows call for more reliable integration methods. Nowadays, artificial intelligence tools such as fuzzy approaches and neural networks have been applied to data fusion problems and resulted in higher fusion accuracy. A simple idea of the AIS and OTHR data fusion based on the fuzzy sets was brought forward in [12] and used in handling inaccuracy when computing association grades for different vectors. The fuzzy C-means clustering method was used in [13] for correlating tracks without merging them. Neural networks were proposed for improvement of AIS and OTHR data fusion [14,15], but the methods employed were rather complicated. In general, these approaches neglected ships which were either not equipped with AIS or unwilling to emit AIS information. Furthermore, the validity of the presented approaches was checked against research data without considering the latency of AIS messages.

In this work, we present a fuzzy-based method for addressing imprecision characterized by these types of sensors. The proposed approach offers better insight in numerical processing than neural networks. AIS and OTHR tracks are loaded into a relational database in order to perform advanced database operations such as data cleaning, filtering and record matching. Actually, FFDs have been used to define data integration constraints among two databases because they take similarity into account. Therefore, to address the development of fusion method, it is necessary to prepare data for processing and analyze different challenges appearing in real scenarios. This approach provides complete picture of maritime situation enabling the detection of possible threats to maritime nation’s interests in EEZ, as well as better insight in events within it. The performance of the introduced method is experimentally tested and discussed. It is worth noting that this paper relies on concepts described in [11], but utilizes a different decision-making algorithm based on fuzzy concept.

This article is structured as follows. In Section 2 the research background is described. Section 3 presents methods of measuring the similarity between AIS and OTHR records. Section 4 gives the algorithm overview and implementation steps. Experimental results are discussed in Section 5. Finally, the paper ends with the conclusions presented in Section 6.

## 2. Background

In this section we considered functional dependencies (FDs), which are a basic concept for data fusion and data cleaning, between AIS records and OTHR tracks. Functional dependencies are one of the fundamentals of Codd’s relational model used as a tool for the database design based on the normalization theory and redundancy elimination. An interpretation of FD, denoted by *X* → *Y*, in the relation *R* defined over the set of attributes *attr*(*R*) is the following: if two tuples *t*_1_ and *t*_2_ have the same value on the attribute *X*, then they also have the same value on attribute *Y*. It reads as *X* determines *Y* or *Y* is functionally dependent on *X*. But, in many practical problems data are not strictly equal. For example, OTHR by its nature involves some level of inaccuracy. In our case, it measures the range, azimuth and radial speed of targets with accuracy expressed in terms of the corresponding resolution cells. In this work, the range resolution cell of OTHR is 1.5 km, angular resolution is 10° and the radial velocity resolution is 0.32 m/s. The accuracy of measurement quantities is in the range of 0.5 to 1.5 respective resolution cells. Matching the AIS and OTHR data means that we have to find similar records. One of the challenging problems in our work is measuring similarities among AIS records and OTHR tracks considering their respective attributes. The main idea is to find the intensity of similarity in order to detect the same targets and possible threats. This assumption leads us to extending FDs to fuzzy functional dependencies. The equality relation used in the above canonical FDs is replaced by a similarity or proximity relation in FFDs as a measure of closeness [16]. The extension enables the specification of new application areas such as data fusion of AIS and OTHR. Today, FDs defined in various ways [17] are not used only for database design purposes, but also in other challenging applications such as data cleaning [18], record matching [19], query relaxation [20], and knowledge discovery [21].

In order to impose fuzzy data dependency, we introduce the definition of FFD *X* $\overset{\theta }{\to }$ *Y* [22]:

min *I* (≈(*t*_1_(*X*), *t*_2_(*X*), ≈ (*t*_1_(*Y*), *t*_2_(*Y*)) ≥ *θ* (1)

where *t*_1_, *t*_2_ ∈ *R* (values of tuples *t*_1_ and *t*_2_ for attributes *X* and *Y* respectively in relation *R*), “≈” is the closeness measure, *I* is a fuzzy implications operator, min denotes the “*and*” operator and *θ* ∈ [0, 1] is the strength of the dependency. The definition of FFD states that if *t*_1_*(X)* is similar to *t*_2_*(X)* then *t*_1_*(Y)* is also similar to *t*_2_*(Y)* with the strength of dependency *θ*, where *X*, *Y* are two sets of attributes in *R*, *X*, *Y* ⊆ *attr*(*R*). Different similarity functions are employed to compare values of considered attributes. In the last two decades several definitions of FFDs have been introduced [23]. Recently, a new method has been developed for computing FFDs by [24].

In case of AIS and OTHR data fusion, *t*_1_ and *t*_2_ are records representing AIS object and OTHR track respectively. Record matching is performed by means of FFD, i.e., a fuzzy measure on [0, 1] is used to compute how similar the AIS object and an OTHR track are, considering common attributes from the corresponding databases and their aggregation. The first step is calculating closeness of the considered attributes using the defined similarity measures. The strength of dependency *θ* is a real number within the range [0, 1], describing the degree of similarity between an AIS object and an OTHR track. In order to detect the parameter *θ*, an aggregation of similarities per observed attributes is applied. The average aggregation function is suggested for calculating the strength of the dependency among AIS and OTHR records. Additionally, this function can provide flexibility in terms of weighting attributes when some of them are more important than others. Thus, in the next section, we will describe a step-by-step procedure for fusion of AIS and OTHR records.

An important aspect related to FD and FFDs is the presence of inference rules enabling the possibility of deriving the new dependencies from the existing ones [25]. This straightforwardly provides an efficient way to derive relations between other attributes in data analysis. These inference rules are based on Armstrong’s axioms. Inference rules must be sound (rules generate valid dependencies) and complete (valid dependencies can be generated by only these rules). For example, we list the inference rules for FFDs (analogously to FDs):

1. Inclusive rule: If *X* $\overset{\theta_{1}}{\to }$ *Y* holds, and *θ*_1_ ≥ *θ*_2_, then *X* $\overset{\theta_{2}}{\to }$ *Y* holds.
2. Reflexive rule: If *Y* ⊆ *X*, then *X* $\overset{}{\to }$ *Y* holds.
3. Augmentation rule: {*X* $\overset{\theta }{\to }$ *Y*} | = *XZ* $\overset{\theta }{\to }$ *YZ*.
4. Transitivity rule: {*X* $\overset{\theta_{1}}{\to }$ *Y, Y* $\overset{\theta_{2}}{\to }$ *Z*} | = *X* $\overset{\to \min(\theta_{1},\theta_{2})}{\to }$ Z.
5. Union rule: {*X* $\overset{\theta_{1}}{\to }$ *Y, X* $\overset{\theta_{2}}{\to }$ *Z*} | = *X* $\overset{\to \min(\theta_{1},\theta_{2})}{\to }$ YZ.
6. Pseudotransitivity rule: {*X* $\overset{\theta_{1}}{\to }$ *Y, WY* $\overset{\theta_{2}}{\to }$ *Z*} | = *WX* $\overset{\to \min(\theta_{1},\theta_{2})}{\to }$ *Z*.
7. Decomposition rule: If *X* $\overset{\theta }{\to }$ *Y* holds, and *Z* ⊆ *Y*, then *X* $\overset{\theta }{\to }$ *Z* holds.

## 3. Research Methodology

Our methodology described here answers the question as to how OTHR and AIS data are fused. Our main goal is to detect records referring to the same targets in a database. For that purpose, we explore the connection between FFDs and the problem of OTHR tracks and AIS data fusion. By matching records from two databases, we perform data analysis per common attributes in order to fuse related records. As an example, if tuple *t*_1_ representing AIS record and tuple *t*_2_ referring to OTHR track have similar values per common attributes, then they are candidates to represent the same vessel in the observed area. FFDs are used to quantify the closeness or remoteness of OTHR and AIS records. They specify a subset of tuples on which the dependency holds.

Initially, the source databases which need to be integrated have unique identifiers. The primary keys for OTHR tracks and AIS data are *ID_Number* and *Maritime Mobile Service Identity* (MMSI), respectively [26]. The use of primary keys eases matching as compared to a situation when alternative attributes need to be found in databases without unique identifiers. Obviously, there is FD between unique identifiers and other attributes in the source databases: *ID_Number* → *A*, and *MMSI* → *B*, where *A*, *B* are sets of attributes in source relations *r*_1_ and *r*_2_. However, in the case of OTHR and AIS data integration there is a FFD (*ID_Number*, *MMSI*) $\overset{\theta }{\to }$ *C*. where (*ID_Number*, *MMSI*) is a composite key (integration pair), *θ* parameter reflecting degree of similarity and *C* is set of common attributes in source relations: *Timestamp*, *Latitude*, *Longitude*, *Velocity* and *Ship Course*. This FFD is examined through the matching process between AIS and OTHR records in order to evaluate the closeness of observed tuples. Due to the inaccuracy and the inconsistency of data provided by OTHR and AIS platforms, we evaluate the closeness of records in order to detect FFDs which are possible candidates for associating related targets.

The parameter *θ* (the strength of fuzzy functional dependency) is calculated as follows:

(2) $$\theta =\frac{1}{5}\left(c_{time}+c_{long}+c_{lat}+c_{vel}+c_{course}\right)$$

In fact, for each common attribute we calculate how close two attribute values are. Then, we aggregate and normalize the closeness values of common attributes. The records from OTHR and AIS source databases with the highest *θ* value are candidates for target association. In order to calculate the closeness values *c* for the attributes *Time* (*c_time_*), *Longitude* (*c_long_*), *Latitude* (*c_lat_*), *Velocity* (*c_vel_*) and *Course* (*c_course_*) the following equations are used:

(3) $$c_{vel}=1-\frac{\left|v_{AIS}-v_{OTHR}\right|}{v_{max}}$$

where *v_AIS_* and *v_OTHR_* represent the target’s speed reported by AIS and OTHR networks respectively, while *v_AIS_* − *v_OTHR_* is their difference. The variable *v_max_* denotes the maximum speed of target communicated by OTHR and AIS systems, i.e., *v_max_* = max ($v_{AIS}^{max}$*,* $v_{OTHR}^{max}$). *c_vel_* represents the speed matching value for the corresponding records from AIS and OTHR databases.

(4) $$c_{course}=1-\frac{\left|C_{AIS}-C_{OTHR}\right|}{C_{max}}$$

where *C_AIS_* and *C_OTHR_* represent the target’s course reported by AIS and OTHR networks respectively, *C_AIS_ − C_OTHR_* is their difference and *Cmax* is maximum course provided for all the targets by OTHR and AIS databases, *Cmax* = max($C_{AIS}^{max}$, $C_{OTHR}^{max}$). *c_course_* is the matching coefficient for the course attribute between two attribute values representing records from AIS and OTHR.

(5) $$c_{long}=1-\frac{\left|Long_{AIS}-Long_{OTHR}\right|}{Long_{max}}$$

(6) $$c_{lat}=1-\frac{\left|Lat_{AIS}-Lat_{OTHR}\right|}{Lat_{max}}$$

where *Long_AIS_* (*Lat_AIS_*) and *Long_OTHR_* (*Lat_OTHR_*) represent the target’s longitude (latitude) reported by AIS and OTHR networks respectively, *Long_AIS_* − *Long_OTHR_* (*Lat_AIS_* − *Lat_OTHR_*) is their difference and *Long_max_* (*Lat_max_*) is maximum value of longitude (latitude) in OTHR and AIS databases. Longitude and latitude are expressed as decimal values (degrees). Closeness between AIS and OTHR records for longitude (latitude) position is shown as *c_long_* (*c_lat_*).

(7) $$c_{time}=1-\frac{\left|t_{AIS}-t_{OTHR}\right|}{t_{interval}(sec)}$$

where *t_AIS_* and *t_OTHR_* represent timestamps of data creation by AIS and OTHR sensors respectively and *t_AIS_* − *t_OTH_*_R_ is their difference. Timestamps *t_AIS_* and *t_OTHR_* are converted to seconds due to efficient computation. The interval of time (*t_interval_*) represents the length of the observed time frame and it is also expressed in seconds. The interval is measured as time duration between two AIS messages.

Presented processing logic is triggered by newly received AIS message. We suppose that we operate with fairly precise AIS data. Furthermore, for every AIS record OTHR candidates are ranked by strength of dependency *θ* in the record matching process. This procedure is shown in the next Section.

## 4. Algorithm

**Figure 1:**
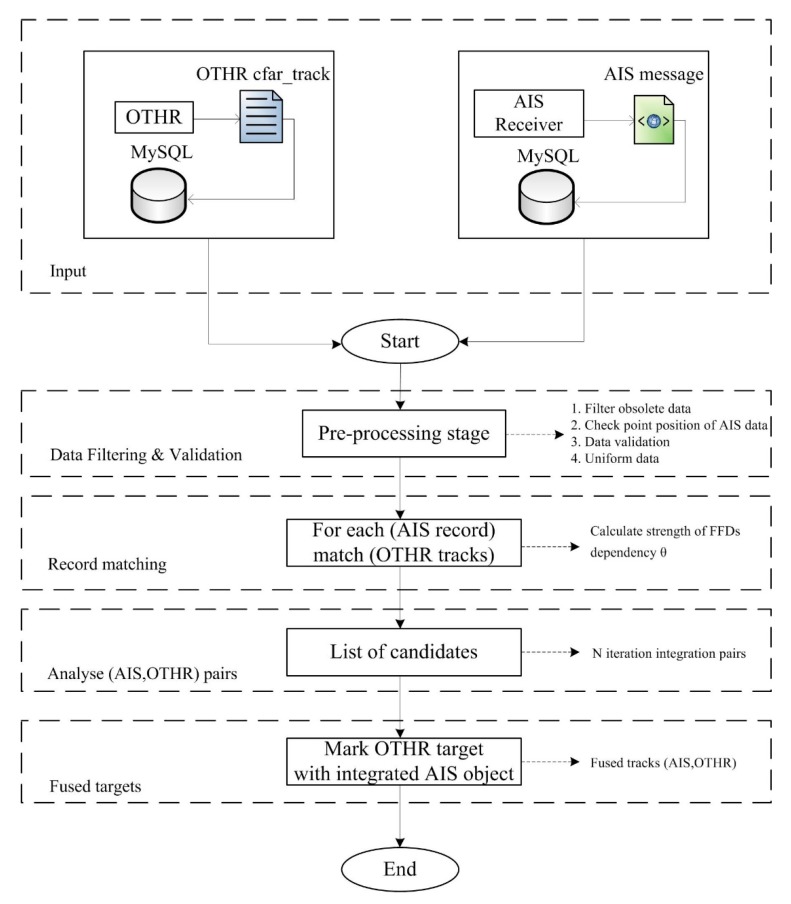
Algorithm for the fusion of automatic identification system (AIS) data and over-the-horizon radar (OTHR) tracks.

In terms of data fusion from AIS and OTHR sensors, we present an algorithm based on aforementioned methodology. This algorithm with steps describing record matching is shown in Figure 1.

Algorithm initialization is triggered by the reception of AIS message. OTHR dataflow is periodic with repetition cycle of 33 s, while AIS system in this geographical area does not provide consistent message delivery. This is particularly the case with Satellite AIS transmissions where latency is even measured in hours. OTHR tracks representing the paths of targets must be stored in the database in order to perform record matching with AIS data. Sometimes, it can be a repository with a long history due to high latency of AIS message delivery. History of OTHR tracks helps in overcoming this issue. The raw messages of AIS and OTHR networks (XML and text file respectively) are converted and imported into MySQL databases. This approach allows efficient data manipulation, storing and flexible searching of data by time, position or target ID. Data is organized into tables according to OTHR and AIS sensors. Both tables have attributes related to target ID (*ID_Number* for OTHR and *MMSI* for AIS), position (*Longitude* and *Latitude*), *Course*, *Velocity* and *Timestamp* of data creation.

After the data is loaded and stored into databases, the first step is pre-processing of OTHR tracks and AIS measurements. This stage involves data filtering and validation for the purpose of completing the state of information. First, it includes data filtering by time. When a new AIS message is received, algorithm deletes old and unneeded OTHR tracks (older than the oldest AIS record and beyond the message time frame). Second, OTHR covers a static (fixed) area, while the AIS continuously changes its covering area. This pre-processing activity is focused on filtering AIS records that are outside the coverage area of OTHR. It is based on geographical coordinates of the data (latitude and longitude). Third, in order to avoid matching of possibly false targets with real AIS data, the Tracker Confidence attribute in OTHR database is used as a criterion for elimination of unconfirmed targets. Tracker Confidence attribute describes reliability of OTHR data [11]. Then data validation is also done over filtered records due to possibility of several detections for the same target in OTHR and AIS databases. Because of data validation, additional logic is applied for analyzed tracks (i.e., OTHR targets with velocity exceeding maximal possible speed of movement (>30 m/s) are excluded). Finally, algorithm performs the data uniformity task. This means that reported radial velocity and azimuth (angle radar—target relative to true north (TN)) registered with OTHR sensor cannot be used for fusion. For that purpose, we use approximations of target course and velocity of OTHR tracks in order to use the same units of measurement for record matching with AIS data. In addition, timestamps are converted to seconds due to closeness calculation. All aforementioned steps are performed over MySQL databases using data extraction queries. The advantage of storing data in relational form is simplicity of data manipulation, keeping of long history of OTHR tracks and further processing.

In the second step, processing logic based on FFDs is applied in the pre-processed tables. Actually, the algorithm searches for a set of possible associating candidates for each AIS point (record). The parameter *θ* is calculated using Equation (2). In this way, for every AIS point we compute a list of candidates for integration. Finally, for every AIS record OTHR integration candidates are ranked by the strength of the dependency *θ*. We determined experimentally the threshold value of the parameter *θ* for finding AIS-OTHR pairs. The criteria of *θ* > 0.80 is applied.

Next step analyses results obtained in the previous activity: the association is done for a given pair of tracks based on the highest value of the parameter *θ*. The previous step is repeated for every new AIS message. If a pair of AIS and OTHR tracks within N cycles is found, then the algorithm confirms the association and passes the pair for fusion to it. Note that, despite the AIS point being treated as fused point, on every new appearance of AIS data, record matching and analyses of OTHR track candidates are repeated. This allows corrections in integration process and better resolution of difficulties and confusing situations (i.e., when multiple targets are close to each other).

In the last step, fused targets are marked as integrated pairs (*ID_Number, MMSI*). Association with an OTHR track is declared and AIS point is treated as the fused point.

## 5. Experiment and Discussion

**Figure 2:**
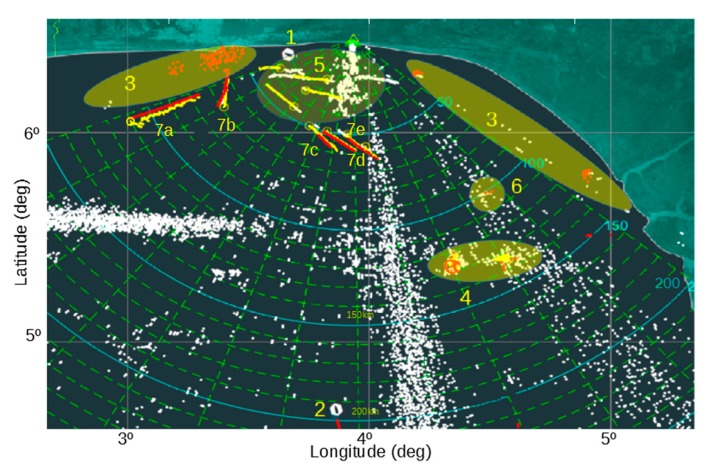
Maritime situation of the OTHR coverage area with reported AIS data in the Gulf of Guinea site (yellow—OTHR targets, red—AIS points, white—radar clutter).

In this section, the experimental results of the above proposed algorithm for data fusion between AIS and OTHR sensors are shown. As described in previous sections, the algorithm is triggered by reception of AIS message. Then the time frame of the AIS message is analyzed and data pre-processing is done in accordance with the steps for data filtering, validation and uniformity. It means that unnecessary (filtering by time) OTHR tracks are eliminated, AIS points outside the radar coverage area are not considered. Due to the absence of a strong regulatory institution, data provided by AIS can be of questionable quality (i.e., there are duplicate entries for the same targets). Before all aforementioned actions, we illustrate a picture of OTHR tracks and AIS targets as shown in Figure 2.

**Figure 3:**
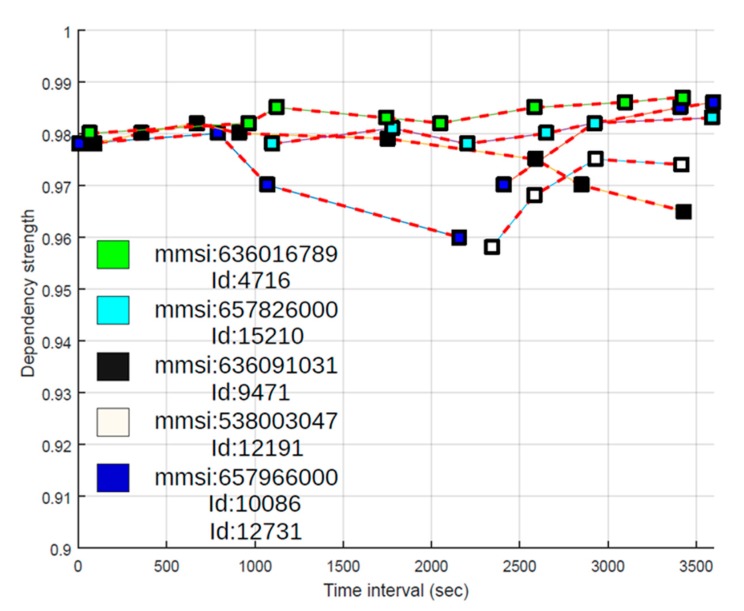
Results obtained for targets association over repeated iterations. Time interval from 16:33 h to 17:33 (3600 s).

In Figure 2, the following situations can be recognized:

1. Target leaving the radar coverage area;
2. Target entering the radar coverage area;
3. AIS data outside of the radar coverage area;
4. Multiple targets reported by AIS inside of one radar resolution cell. This situation is quite regular since resolution cell can be very large in comparison to the vessel size. In the presented scenario’ two oil platforms with all corresponding vessels near them are shown. In practice, we mark these zones for a better understanding of maritime situation;
5. Vessels detected by OTHR, but not transmitting AIS data. Although this situation is rare in developed countries, in the Gulf of Guinee it is quite common and can present a security threat;
6. Vessels transmitting AIS data, but not detected by OTHR. This situation usually occurs when a smaller vessel delivers AIS data, although it may also present OTHR’s missed target (there is no sensor with 100% detection probability);
7. Vessels labeled as 7a, 7b, 7c, 7d and 7e in the radar coverage area represent successful data fusion between AIS and OTHR sensors. Fusion parameters are calculated for them and shown in Figure 3.

The white points in Figure 2 are potential detections generated by OTHR. A large number of detections is a consequence of a complex environment in which OTHR operates, which contains high amounts of radar clutter. A high amount of clutter causes a large number of false detections and possibly missed detections of real radar targets. In our case, the sea swell is the main cause of radar clutter seen between 5 and 40 km away, at azimuth angles of ±10°. External radio sources such as Radio Frequency Interference (RFI) are also seen in two directions: −20° and −45°. A group of false detections comes from ionospheric interference, 10 km to 20 km wide, at the latitude of about 5.5°, covering azimuth angles from 10° to 60°. The clutter elimination (filtering) is performed during the pre-processing step.

As an example, a vessel labeled as 7a in the Figure 2. will be used to demonstrate the fusion process. In order to calculate the dependency strength *θ*, it is necessary to predict velocity and course of OTHR tracks using approximation and average measurements. OTHR target speed is approximated based on the difference in the distance registered by the radar and the period of time detection (Δ*t* = 33 s) for the two neighboring positions. Projected OTHR target velocity is equal to the average value of the approximate speed measures over an observed time frame. Approximation of the course for OTHR ships is calculated by using WGS84 terrestrial reference system. To demonstrate the calculation of the dependency strength *θ*, let us consider the following AIS and OTHR records given in Table 1 and Table 2 representing targets in Figure 2.

Using Equations (3)–(7) we calculate closeness between attributes of AIS target and OTHR track inside a defined time frame as shown in Table 3:

Finally, we compute the dependency strength of the FFD between records by using Equation (8):

(8) $$\theta =\frac{c_{time}+c_{long}+c_{lat}+c_{vel}+c_{course}}{5}=\frac{4.9127}{5}=0.9825$$

Iterating through all OTHR records and matching with pointed AIS target, we get a pair (*ID_Number*, *MMSI*) for possible association (the highest value of parameter *θ*). In our case, AIS target with *MMSI* = 636,091,031 and OTHR track with *ID_Number* = 9471 are candidates for fusion. In order to increase the possibility of correct matching, we repeat the procedure described for newly received AIS message and calculate the dependency strength in this iteration as well. The results of matching for the fused pairs or targets within a time frame of 1 h (from 16:33 h to 17:33 h) are shown in Figure 3.

**Figure 4:**
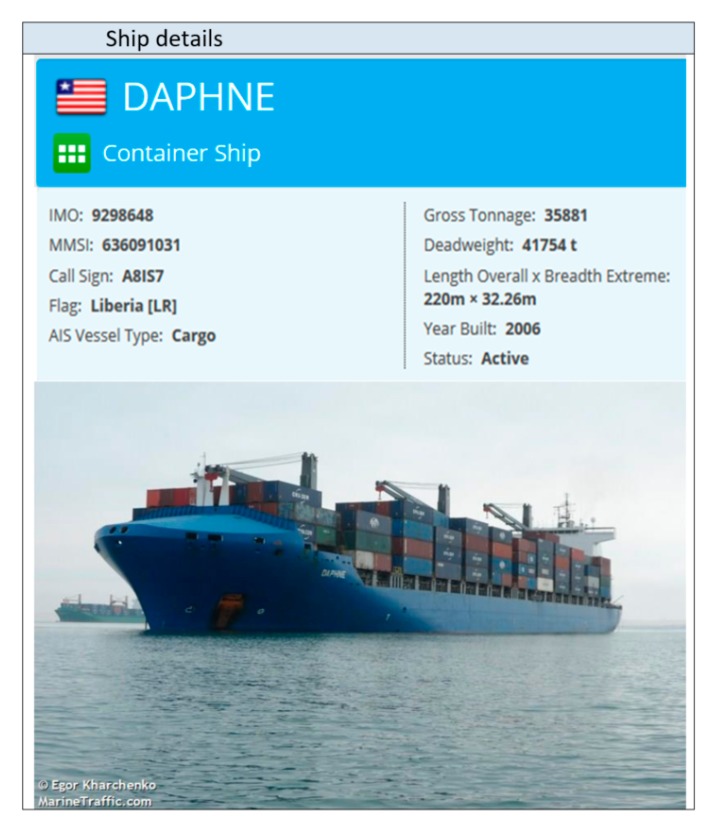
Fused AIS point with OTHR track—Dafne ex Emirates Asante [27].

In the described experiment, there were 20+ vessels with AIS data and 13 OTHR targets. After AIS data outside of OTHR coverage area were filtered out, only 13 vessels reported by AIS remained for possible fusion. Results of data fusion for vessels labeled as 7a, 7b, 7c, 7d and 7e are depicted in Figure 3. Time axis provides more informative maritime picture. By using the MMSI code it is possible to obtain detailed information about the ship [27] as shown in Figure 4. It is important to note that cases 7b and 7c from Figure 2 are also represented as recognized targets and all the data are fused accordingly despite a significant AIS latency (7c) and later OTHR detection (7b). This is illustrated in Figure 3 where AIS message latency of a detected vessel is indicative (cyan squares) and first OTHR detections of vessel labeled as 7b were received long after the reception of AIS data (white squares). It is interesting to consider the fusion in the case of vessel labeled as 7e. The AIS target is fused with two different OTHR tracks due to the interruption of OTHR detection (blue squares in Figure 3). Since all objects around oil platforms are detected by OTHR as one, the OTHR track can be fused with only one AIS with the most likely attributes, while the other AIS will remain unfused. This should be marked for the operators of the maritime monitoring system.

**Figure 5:**
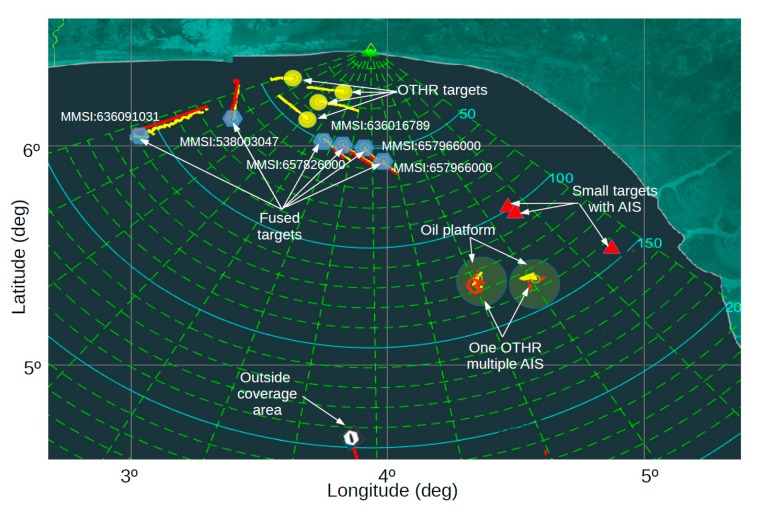
The results of experiment—complete operational picture for maritime surveillance (yellow—OTHR targets, red—AIS points, blue—fused data).

AIS data that cannot be associated with any of the OTHR tracks will also stay unfused as well as OTHR tracks which cannot be associated with any AIS data (non-cooperative targets that pose potential threats). After all processing is done, complete operational picture in the OTHR coverage area is shown in Figure 5. It is worth mentioning that maritime situation presented in Figure 5 is formed after 4 h of collecting and processing data delivered by available sensors.

There are critical and confusing situations such as when multiple targets are close to each other, which present a real challenge for any fusion procedure. In our work, we suggest an algorithm designed to autocorrect faulty AIS-OTHR fusion candidates through repeated iterations. This autocorrection happens when targets are moving away from each other. On the other hand, when there are multiple non-moving targets in small areas, usually in front of harbors and oil platforms, the finite resolution of OTHR prevents proper detection of vessels. Operators usually consider such OTHR detections as redundant or unnecessary. These areas are marked as illustrated in Figure 5 and often excluded from OTHR tracking. Furthermore, the correctness of fusion can be tested by estimating the AIS target’s radar cross-section parameter, based on its position and orientation with respect to OTHR, as well as the ship size information, available from third-party sources. By comparing the measured Signal-to-Noise (SNR) of OTHR detection with pre-set experimental tables, it is possible to estimate the ship size class and perhaps to resolve to which AIS target the observed OTHR track is most likely to be associated. Thus, intensive research will be needed in future.

Figure 5 illustrates a complete maritime picture at OTH distances in the OTHR coverage area.

## 6. Conclusions

In this article, a method of fusing information obtained from AIS and OTHR sensors based on the fuzzy functional dependency approach has been presented. We describe a computational algorithm that focuses on the fuzzy decision making technique. It associates OTHR target tracks with available AIS ID records within the observed message time frame. Actually, the algorithm computes the record matching based on fuzzy dependency between AIS data and OTHR tracks. As shown, a number of iterations which take into account the similarity between database tuples, increases the odds for correct data fusion. This novel fuzzy-based method reveals the complementarity of two different sensors and enhances their integration. The integrated information provides a more informative maritime situational awareness. The reception of the AIS message is unsteady, especially in case of satellite transmission, causing difficulty in data fusion and analysis, as well as missing information. OTHR tracks not being fused with any AIS data can be identified as non-cooperative targets. Identification of these targets is a matter of future consideration. The performance of the proposed algorithm has been evaluated using real data. The experiment has shown promising results.

Future work is intended for applying this method to a larger-scale application problem (multiple OTHRs and multiple AIS sources covering a much larger geographical area). In addition, the application of decision-making theory is recognized as a possible direction for the method improvement.

## Figures and Tables

**Table 1:** AIS point.

MMSI	UTC Time	Latitude (deg)	Longitude (deg)	Course (deg)	Velocity (m/s)
636,091,031	16:48:10 (60,490 s)	6.142435	3.229788	247.1	9.36

**Table 2:** OTHR track.

ID_Number	UTC Time	Latitude (deg)	Longitude (deg)	Course (deg)	Velocity (m/s)
9471	16:48:05 (60,485 s)	6.128810	3.234660	248.68	9.77

**Table 3:** Closeness between AIS and OTHR attribute values.

c_time_	c_lat_	c_long_	c_course_	c_vel_
0.9986	0.9979	0.999	0.9582	0.959
